# Mother knows best: nest-site choice homogenizes embryo thermal environments among populations in a widespread ectotherm

**DOI:** 10.1098/rstb.2022.0155

**Published:** 2023-08-28

**Authors:** Brooke L. Bodensteiner, John B. Iverson, Carter A. Lea, Carrie L. Milne-Zelman, Timothy S. Mitchell, Jeanine M. Refsnider, Kameron Voves, Daniel A. Warner, Fredric J. Janzen

**Affiliations:** ^1^ Department of Ecology and Evolutionary Biology, Yale University, New Haven, CT 06511, USA; ^2^ Department of Ecology, Evolution, and Organismal Biology, Iowa State University, Ames, IA 50011, USA; ^3^ Department of Biology, Earlham College, Richmond, IN 60071, USA; ^4^ Office of Research Proposal Development, Tulane University, New Orleans, LA 70118, USA; ^5^ Department of Biology, Joliet Junior College, Joliet, IL 60431, USA; ^6^ College of Biological Sciences, University of Minnesota, St. Paul, MN 55108, USA; ^7^ Department of Environmental Sciences, University of Toledo, Toledo, OH 43606, USA; ^8^ Archbold Biological Station, Venus, FL 33960, USA; ^9^ Department of Biological Sciences, Auburn University, Auburn, AL 36849, USA; ^10^ Kellogg Biological Station, Michigan State University, Hickory Corners, MI 49060, USA

**Keywords:** climate change, local adaptation, maternal effects, nest-site choice, phenotypic plasticity, Bogert effect

## Abstract

Species with large geographical ranges provide an excellent model for studying how different populations respond to dissimilar local conditions, particularly with respect to variation in climate. Maternal effects, such as nest-site choice greatly affect offspring phenotypes and survival. Thus, maternal behaviour has the potential to mitigate the effects of divergent climatic conditions across a species' range. We delineated natural nesting areas of six populations of painted turtles (*Chrysemys picta*) that span a broad latitudinal range and quantified spatial and temporal variation in nest characteristics. To quantify microhabitats available for females to choose, we also identified sites within the nesting area of each location that were representative of available thermal microhabitats. Across the range, females nested non-randomly and targeted microhabitats that generally had less canopy cover and thus higher nest temperatures. Nest microhabitats differed among locations but did not predictably vary with latitude or historic mean air temperature during embryonic development. In conjunction with other studies of these populations, our results suggest that nest-site choice is homogenizing nest environments, which buffers embryos from thermally induced selection and could slow embryonic evolution. Thus, although effective at a macroclimatic scale, nest-site choice is unlikely to compensate for novel stressors that rapidly increase local temperatures.

This article is part of the theme issue ‘The evolutionary ecology of nests: a cross-taxon approach’.

## 1. Introduction

Species with environmentally sensitive traits and large geographical ranges are excellent study systems for answering questions about local adaptation and/or phenotypic plasticity [1–4]. Adaptation and phenotypic plasticity might permit organisms to persist in spatially and temporally changing environments ([5,6], but see [7]). Of major concern are the biological impacts of climate warming, as a 1.5°C increase in global surface temperatures is conservatively predicted by 2100 [8]. An appropriate system to address urgent questions associated with *in situ* biotic responses to climate change, particularly temperature, requires three critical characteristics: (i) the system must experience extensive variation in temperatures; this system should also (ii) contain at least one trait directly impacted by temperature that is (iii) fundamentally tied to critical processes associated with fitness.

Owing to the direct linkage of temperature via thermally mediated biochemical reactions, studies of physiological and morphological ecology have taken centre stage in contributing to our understanding of evolutionary responses to changing thermal environments ([5,9] but see [7]). However, it has also become increasingly evident that behavioural responses to local environmental change could alter the evolutionary trajectory of physiological and morphological traits. A factor often overlooked while determining organismal responses to variation across a wide geographical range or susceptibility to a rapidly changing climate is behaviour, particularly if behaviour transpires before an organism is born. In egg-laying organisms, the maternal behaviour of nest-site choice is particularly interesting because it has ecological and evolutionary consequences for both mother and offspring [10,11]. In the broadest sense, nest-site choice is a maternal effect by which females can indirectly affect the survival and phenotype of their offspring [12–19]. Nest-site choice is known to influence a variety of biotic and abiotic factors that can impact developing offspring across taxa: hydric conditions [20,21], predation risk [22,23], light conditions [24], pH [20] and temperature [25–31].

However, it has also become increasingly evident that behavioural responses to local environmental change could alter the evolutionary trajectory of physiological and morphological traits. The phenomenon in which regulatory behaviours (e.g. thermoregulation or nest-site choice) dampen selection and slow evolution by shielding organisms from environmental variation is referred to as the Bogert effect [4,32,33]. Work on this matter has largely focused on the intraspecific evolutionary ecology of local populations of short-lived organisms that vary in altitude (e.g. *Anolis* lizards in [34] and *Phrynosoma hernandesi* in [35]). The extent to which the Bogert effect manifests across generational boundaries and in species with long lifespans that occupy large geographical ranges with minimal gene flow remains unknown. Theoretically, such taxa should be more prone to exhibiting phenotypic plasticity as an immediate response to selection under rapidly changing conditions, given their long generation times [36,37].

**Figure 1.**
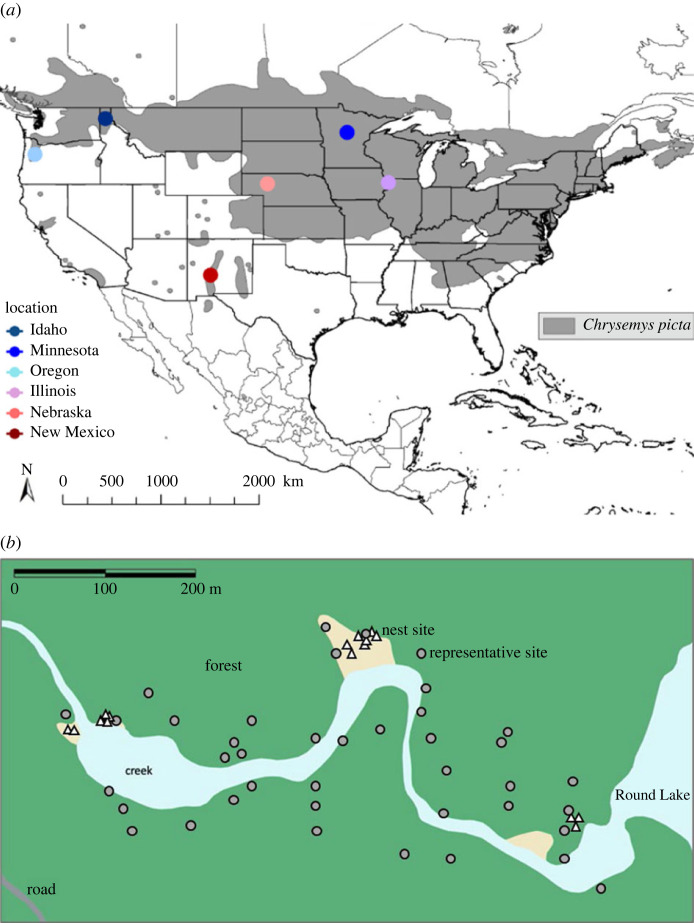
(*a*) Map of *Chrysemys picta* distribution in grey and field sites colour coded by latitude, with higher latitudes in cooler colours and lower latitudes in warmer colours. (*b*) Map of Idaho study location, depicting natural nests (triangles) and our representative sites (circles). The green signifies forest and the cream colour specifies areas of sand. Modified from [30].

The painted turtle, *Chrysemys picta,* is notably conducive to investigating adaptive responses to changing thermal environments. First, painted turtles are geographically widespread (inhabiting approx. 4.1 million km^2^; [38]) ranging from the Atlantic to Pacific Oceans in North America and from Canada to Mexico ([39,40]; figure 1). Thus, different parts of the range experience divergent climatic conditions. Second, incubation temperature affects multiple offspring phenotypes (e.g. growth) [41–44]. Painted turtles also exhibit temperature-dependent sex determination (TSD), such that females are produced at warmer incubation temperatures and males at cooler temperatures (e.g. [45]). Experiments have demonstrated the extreme sensitivity of this classic polyphenism; changes in incubation temperature over only a few degrees switch production from entirely males to entirely females [46,47]. Third, many temperature-sensitive phenotypes (e.g. body size and sex) in this long-lived turtle are related to organismal fitness and population demography [26,45,48].

Given their broad geographical (and thermal) range and extreme thermal sensitivity during incubation, the painted turtle is an excellent model to explore local adaptation of embryos to temperature. Recent work on this species explored the extent to which egg physiological ecology is locally adapted to thermal conditions [44,47]. Despite theoretical expectations (e.g. [36]), no latitudinal gradients in key offspring traits (e.g. incubation duration and body size) have been detected [44,47]. This lack of local adaptation is potentially explained by the Bogert effect (*sensu* [4]), because reptile embryos have a very limited ability to actively compensate for, or avoid, unfavourable nest thermal conditions [49]. Adult females may mitigate exposure of their developing embryos in these populations to divergent thermal conditions by nesting in microhabitats with particular thermal characteristics (e.g. shadier, cooler nests; [43,50]). Consistent with this view, a common-garden field experiment of nesting behaviour demonstrated that painted turtles from across the range retained differences in nesting phenology and nest depth, but exhibited almost complete plasticity in choice of canopy cover over nests at oviposition [51]. Whether the Bogert effect is operational in a cross-generational manner, in which the mother's behaviour shields her offspring from selection and therefore stymies evolution in free-ranging populations, remains an open question.

The goals of our study were to examine nest-site choice and its thermal consequences by characterizing nest microhabitats—focusing on canopy cover at oviposition (*sensu* [52]) and its relationship to nest thermal conditions during the crucial organogenetic period of embryonic development—at both local and broad geographical scales and through time. Specifically, we addressed the following questions: (i) how do natural nests differ from other potential nest sites in each study location? (ii) how do natural nests differ among study locations across a wide geographical range? and (iii) do key hatchling traits (mass and carapace length) vary predictably across latitudes in natural nests? Owing to the fact that the thermal reaction norms for embryos from these different locations (populations) are not explained by geography [44,47], we hypothesize that turtles from all locations target the same narrow range of temperatures for their nests which we can quantify by measuring nest temperatures at different locations. We anticipate that turtles use available microhabitat differently across locations to compensate for macroclimatic variation. For example, at higher latitudes, nesting behaviour may entail selecting the most exposed, warmest sites in contrast with nests sites selected at lower latitudes [53,54]. Again, because developmental thermal reaction norms of offspring from these populations are not related to latitude [44,47], we do not expect a latitudinal pattern related to body size (e.g. Bergmann's rule). However, more generally there is mixed evidence in finding patterns consistent with Bergmann's rule in turtle species [55,56]. This line of questioning may provide support for the Bogert effect acting across generations in these populations (i.e. maternal nesting behaviour may buffer developing embryos from thermally induced selection). Regardless, investigating nesting behaviour and its effects on nest thermal conditions across geographically distant locations of a single species will enrich our understanding of how such widespread taxa maintain successful populations in varied contemporary environments and also provide insights into their vulnerability to future temperature increases.

## 2. Methods

### (a) The study system

We conducted a comparative study of six *C. picta* populations west of the Mississippi River [39,40]. Our study locations (Idaho, Illinois, Minnesota, Nebraska, New Mexico, and Oregon; table 1; figure 1) ranged across approximately 33 degrees of longitude and approximately 15 degrees of latitude. Three of these locations (Illinois, Nebraska and New Mexico) are the foci of long-term studies [52,57,58]. Females in these locations emerge from freshwater habitats primarily in May and June to excavate shallow, Erlenmeyer flask-shaped, subterranean nests with a mean depth of approximately 10 cm [59]. In this species, ovaries are differentiated at constant incubation temperatures above 29°C and testes are differentiated at constant incubation temperatures below 27°C [60]. The temperature-sensitive period of sex determination, and organogenesis more generally, corresponds with the middle third of embryonic development [61,62].

**Table 1.** Comparisons of field localities including: latitude and longitude, mean historic July air temperature 1971–2000, substrate type, shade structures and years of data collection.

name	state	latitude longitude	mean July air temp (°C)	substrate types	shade structures	years of data collection
Round Lake State Park	Idaho	48.16° N	18.3	loam and sand	mostly mature coniferous trees with sparse deciduous trees	2013–2016
		116.64° W				
Tamarac National Wildlife Refuge	Minnesota	46.95° N	20.0	loam and gravel	mature deciduous and coniferous trees; sparse grass prairie	2012; 2014–2015
		95.64° W				
Smith and Bybee Wetland Natural Area	Oregon	45.62° N	20.1	loam and sand	willow thickets and sparse deciduous trees	2014–2016
		122.72° W				
Upper Mississippi River National Wildlife and Fish Refuge	Illinois	41.94° N	22.7	loam, gravel, and sand	mature deciduous and coniferous trees	2013–2016
		90.11° W				
Crescent Lake National Wildlife Refuge	Nebraska	41.76° N	23.4	loam, gravel, and sand	various buildings and mature deciduous trees	2013–2015
		102.43° W				
Bosque del Apache National Wildlife Refuge	New Mexico	33.78° N	25.3	sand and gravel	willow thickets with various grasses and sedges	2014–2016
		106.89° W				

### (b) Field methods

Nesting activities at each study location were surveyed in May and June during three–five nesting/incubation seasons (2012–2016). Surveys were conducted hourly from sunrise to sunset daily. Nesting turtles were monitored from a distance until nest construction was complete. Each nest was carefully excavated to count and weigh eggs, which were returned in the order they were removed; and a data logger (Thermochron® iButton® DS1921G, accuracy/resolution ± 1°C/0.5°C; Maxim Integrated: San Jose, CA) programmed to record temperature hourly and wrapped in latex and Parafilm to provide waterproofing was placed in the middle of the cavity among the eggs. The nest cavity was then backfilled with soil and, except at the Illinois site, protected from predation with 1 cm wire hardware cloth (cut to 25 × 25 cm pieces) and mapped for relocation. To quantify canopy cover above each nest at oviposition, a variable linked to nesting behaviour in prior studies (e.g. [28]), a hemispherical photograph was taken using a Nikon Coolpix 5200 outfitted with a 180° fisheye lens. The camera was placed directly on top of the nest cavity and positioned so the top of the camera faced north. Photographs were uploaded into Gap Light Analyzer software to calculate canopy cover [63,64].

To evaluate nest-site choice, we identified specific sites within the nesting area of each study location that were representative of microhabitats available for nesting. Using geographical information system methodology to create a probability-based spatial sampling design, we generated 40 sets of coordinates within the perimeter of each study location. These 40 coordinates were chosen from a distribution of shade cover percentages found within our delineated nesting area excluding impermeable surfaces and human structures. This was done so we could have a representation of the shade cover available across study locations ([65]; figure 1). These representative sites are analogous to the operative temperature (the null model) in Huey *et al*. [32], illustrating how nest conditions (e.g. shade cover and nest temperatures) of a hypothetical, randomly nesting (null) turtle would change across latitudes (figure 1). After nesting began at a study location, an iButton was placed at mean nest depth (10 cm) and a hemispherical photograph was taken at each of the 40 representative sites, as described above for natural nests. Our dataset included 1821 hemispherical photos and 999 thermal traces across six localities over 5 years. After the incubation period (approx. three months), natural nests were excavated and hatchlings collected. Unhatched eggs and/or egg mortality were noted. Hatchlings were relocated to Iowa State University and phenotypes were then measured.

### (c) Statistical methods

Hemispherical photographs were used for the representative sites to measure canopy cover across all years in this study. Representative site photographs were analysed using the same threshold pixel settings as the natural nests to ensure shade cover comparability between years. Percentage canopy cover at oviposition was arcsine transformed and a Tukey correction was used for multiple comparisons [66].

The middle third of incubation is the primary period of overall organogenesis and corresponds to the period when sex is determined irreversibly by incubation temperatures, which is probably during July in our study populations (e.g. [67]). Thus, hourly nest and representative site temperatures during this month were converted to constant temperature equivalents (CTEs), which facilitate statistical comparisons and interpretations relative to laboratory incubation studies [68–70], but also among nests within locations, across years within locations and across locations. The CTE model translates fluctuating temperatures into values comparable to a constant temperature during incubation, calculating the temperature associated with the median developmental point in *C. picta*. We thus focus on this single downstream example of a potentially phenotypically meaningful thermal metric and its relationship to nest-site choice earlier in the season. We also analyse and report on more traditional minimum, maximum and mean hourly temperatures in July recorded in nests and in representative sites.

Values for the CTE were estimated in Matlab v. R2018a (Mathworks®; Natick, MA), using code provided in Telemeco *et al*. [70]. For each nest and representative site, we used mean diel temperatures and 1/2 diel ranges to estimate daily CTEs, then used the median daily CTE value to represent thermal conditions. The recorded data contained occasional unrealistic values (less than 0°C or greater than 60°C), apparently owing to data logger error. However, temperatures of less than 14°C [71] or greater than 34°C [72] result in zero development, so occasional extreme values would only affect CTE estimates if they shifted the location of the daily median [47]. By contrast, the temperatures experienced at some of the representative sites were consistently too low to facilitate embryonic development. Figures were made using R v. 3.5.0 [73].

Except where otherwise noted, all statistical analyses were performed in SAS version 9.4 (SAS Institute, Cary, NC). A general linear model was used to compare canopy cover at oviposition from hemispherical photos between nest sites and representative sites within locations as well as across locations. Year nested within location and location were treated as fixed effects. July temperatures in nest sites and in representative sites within and across study locations were analysed using a general linear model in the same manner. A general linear model was used to investigate differences between hatchling mass and carapace length across our study locations.

## 3. Results

### (a) Nest characteristics

**Figure 2.**
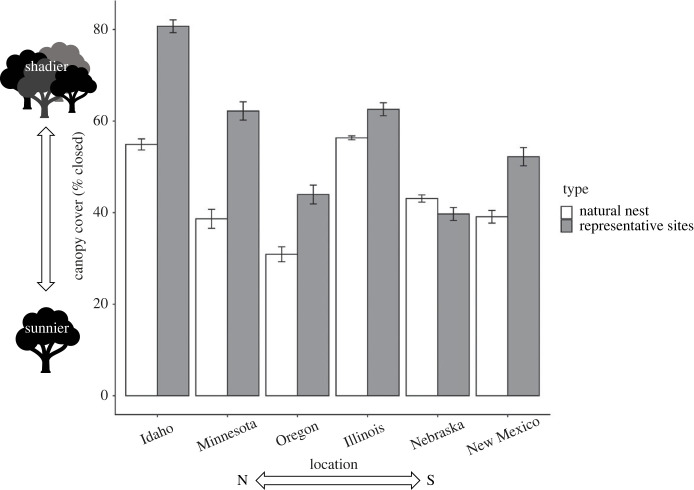
Least-squares means comparisons of per cent canopy cover between natural nests and representative sites across six locations. Nebraska was the only location where there was not a significant difference between natural and representative nest sites. Bars represent 1 s.e. Locations are ordered by latitude moving from north to south from right to left.

Across locations, canopy cover ranged from 1.6% over one representative site in New Mexico to 90.4% over one representative site in Idaho. Overall canopy cover was greater for representative sites versus natural nests (*F*_1,1810_ = 206.59, *p* < 0.0001), and this pattern was evident within each study location except for Nebraska (figure 2). Moreover, the interaction of location and site type (natural versus representative; *F*_5,1810_ = 37.81, *p* < 0.0001) indicated that the difference in canopy cover between site types varied across locations (figure 2). In Idaho, for example, representative sites were 25.8% more shaded than the average Idaho natural nest, whereas in Nebraska, representative sites were only 1.4% less shaded than the average Nebraska natural nest. Nest canopy cover at oviposition differed among locations (*F*_5,1810_ = 147.04, *p* < 0.0001), but did not follow a clear latitudinal pattern. For example, canopy cover at oviposition was similar for nests in locations as far apart as southern New Mexico and northern Minnesota (figure 2).

**Figure 3.**
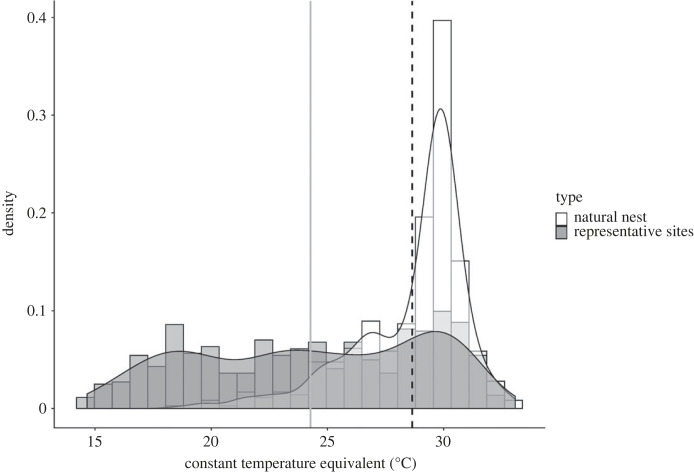
Density plots of the CTEs for the natural nests (white) and the representative sites (grey). The black dashed line represents the mean CTE of natural nest sites and the grey solid line represents the mean CTE of our representative sites.

The range of hourly temperatures was similar between site types. When converted to July CTEs, however, those thermal data revealed that females chose nest sites that averaged more than 4°C warmer than representative sites (28.9 versus 24.7°C; *F*_1,1016_ = 367.79 *p* < 0.0001; figure 3).

**Figure 4.**
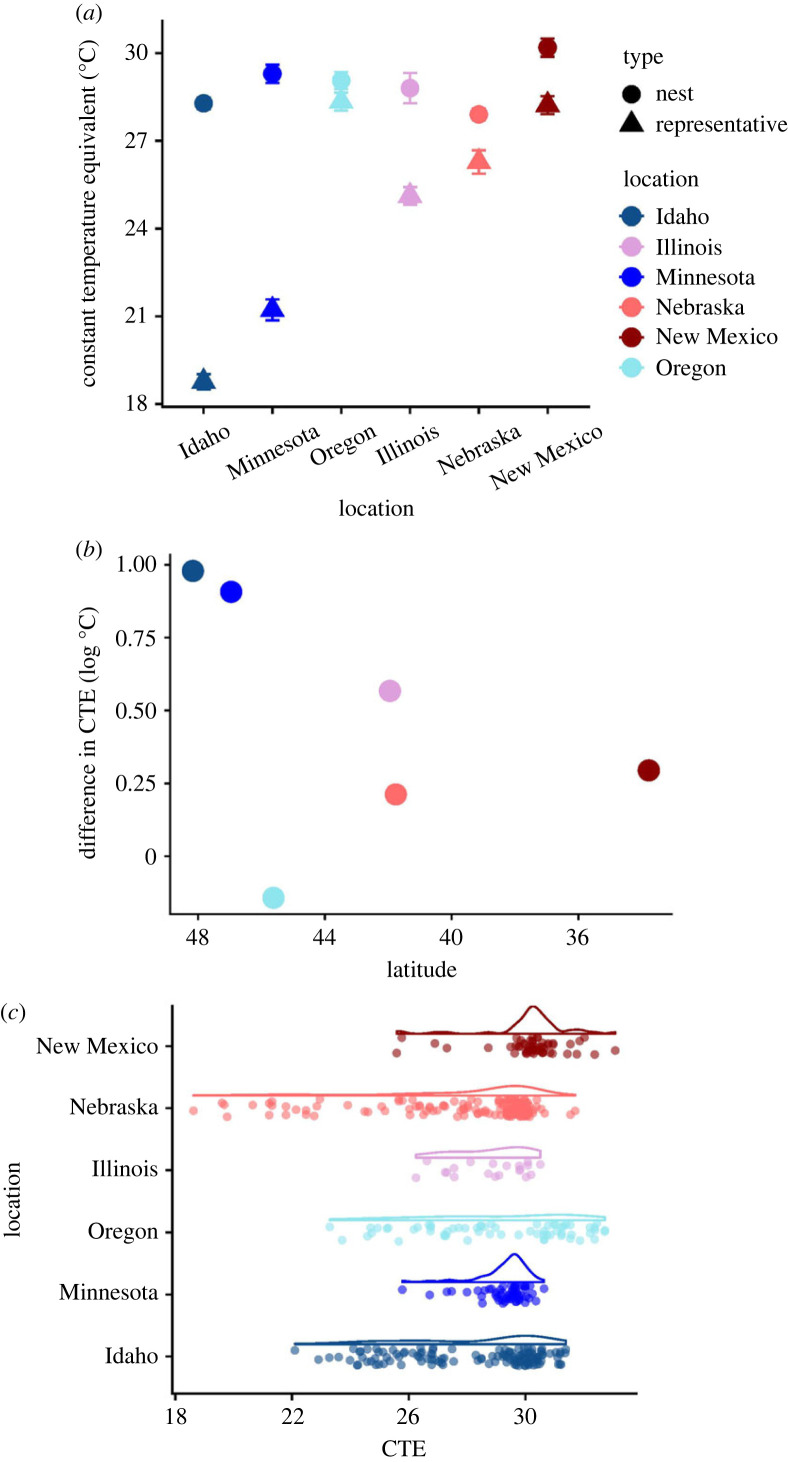
(*a*) Least-squares means comparisons of CTEs (in °C) between natural nests and representative sites across locations. Bars represent 1 s.e. (*b*) The difference in mean CTEs between natural nests and representative sites across latitudes. (*c*) Distributions of CTEs of natural nests across locations. Locations are ordered by latitude moving from north to south from left to right or bottom to top.

The CTEs also differed by location (*F*_5,1016_ = 135.56, *p* < 0.0001), location-specific year (*F*_14,1016_ = 19.25, *p* < 0.0001), and the interaction between location and site type (*F*_5,1016_ = 94.32, *p* < 0.0001); this general pattern held even when looking at the approximate mean, minimum and maximum nest and representative site temperatures (electronic supplementary material, table S1 and figure S1). The magnitude of the differences in CTEs between natural nests and representative sites differed among locations (figure 4). For example, the CTEs in Idaho natural nests were 9.5°C warmer than representative sites, but in Oregon, natural nests were only 0.7°C warmer than their representative sites. Notably, the differences within a study location between CTEs from natural nests and representative sites followed a latitudinal pattern; nest sites from higher latitude populations were substantially warmer than their corresponding representative sites, except in Oregon (figure 4). The July CTEs for natural nests also differed among study locations (*F*_5,446_ = 15.01, *p* < 0.0001) and by location-specific year (*F*_14,446_ = 25.54, *p* < 0.0001; electronic supplementary material, figure S2 and table S2). Comparisons across years within a location revealed a complex temporal pattern in which year often played a major role in influencing nest CTEs (electronic supplementary material, figure S2 and table S2). Among natural nests the canopy cover and nest temperatures (CTEs) within locations revealed complex patterns between the effects of canopy cover and year, in which greater canopy cover does not necessarily equate to cooler nest temperatures ubiquitously (electronic supplementary material, figure S3 and table S3). Factors such as time of day the nest receives the most sunlight, small sample sizes in certain year/location combinations and a majority of nests having a per cent canopy closed value of 30–60% across all locations may be driving the lack of a strong correlation between canopy cover and CTEs.

### (b) Hatchling characteristics

**Figure 5.**
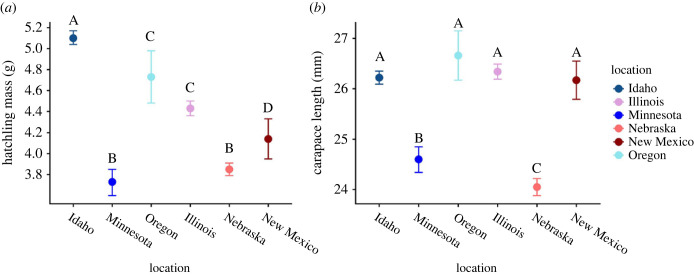
(*a*) Least-squares means comparisons of body mass of hatchlings incubated in natural nests across locations. (*b*) Least-squares means comparisons of carapace length of hatchlings incubated across locations. Letters A-D indicate a significant difference between hatchling mass and carapace length. Bars represent 1 s.e. Locations are ordered by latitude moving from north to south from right to left.

Mass measurements were taken from 1786 hatchlings and carapace length from 1423 hatchlings. Measurements of hatchling mass varied across locations (*F*_5,1,780_ = 200.3, *p* < 0.0001; *R*^2^ = 0.36) but did not follow a latitudinal gradient (figure 5*a*). Similarly, carapace length varied across locations (*F*_5,1,417_ = 116.9, *p* < 0.0001; *R*^2^ = 0.29) but did not follow a latitudinal gradient (figure 5*b*).

## 4. Discussion

Evolutionary adaptation and/or phenotypic plasticity enable populations to persist when environmental conditions change. An important means by which to accomplish these solutions is through maternal effects. We find that, across substantial geographical space and attendant environmental divergence, maternal nesting behaviour in turtles appears to buffer embryos from undesirable local thermal conditions. That is, nest-site choice homogenizes nest temperatures during a crucial period of development and thus shields embryos from otherwise experiencing temperature-induced natural selection. Consequently, latitudinal gradients, widely considered to serve as important signals of adaptation to corresponding latitudinal shifts in environmental conditions [74], are weak at best for key thermally linked offspring traits in this species [44,47]. Thus, maternal effects and phenotypic plasticity might play greater roles than evolutionary adaptation for longer lived organisms than for shorter lived ones in responding to rapid environmental change [36,75].

The microhabitats we assessed—measures of canopy cover and their translation into thermal microenvironments—differed between natural nests and representative sites. Canopy cover was more open at oviposition in maternally selected nest sites, and therefore these sites also were generally warmer in July compared to available local habitat. Only in Nebraska did canopy cover not differ substantively between natural nests and representative sites, but seemingly this is the exception that proves the rule—Nebraska had the most open canopy overall in its nesting area among our study locations (figure 2). In more northern locations, nests tended to have considerably less canopy cover at oviposition and warmer conditions in July compared to representative sites (figures 2 and 4). Consequently, typical nest temperatures in July were remarkably alike among locations, with CTEs varying only approximately 2°C (electronic supplementary material, figure S2) hovering around 28–30°C (figure 4). Meanwhile, the mean July air temperature across these same locations differed by approximately 7°C (table 1). Therefore, females are not just choosing more open nest sites across the geographical range; they appear to be targeting specific nest microhabitats (e.g. [76]). This conclusion accords with a common-garden study in which *C. picta* from many of these same locations chose nest sites with similar canopy cover under semi-natural conditions. The behavioural plasticity in this maternal effect yielded nests with similar thermal environments and, not surprisingly then, similar offspring sex ratios [51].

Of note, the distributions of July thermal data from nests and representative sites indicate that females apparently chose nest sites that buffered thermal minima during development rather than maxima, as hypothesized by other researchers (e.g. [25,77]). Proper development is thermally dependent in turtles, such that embryos do not progress below and above certain temperatures. In *C. picta*, constant incubation temperatures of 14°C and 34°C equate to the developmental thermal minimum and maximum, respectively [70]. No natural nest CTEs were outside the thermal minimum or maximum for development (20.1–30.4°C). By contrast, some representative sites exhibited CTEs that reached the developmental thermal minimum (figure 3). Overall, minimum temperatures were approximately 6°C cooler in representative sites than in actual nests, suggesting that female turtles may buffer nests against excessive cooling and thereby improving the likelihood that embryos can complete development before winter (e.g. [78]).

The CTE range of natural nests importantly encapsulated estimates of the pivotal temperature of sex determination, at which the offspring sex ratio is 1 : 1, for these populations [47]. Yet, because the latitudinal pattern in preference for canopy cover at oviposition was weaker than for nest temperatures in July, maternal behaviours and microhabitat measures that were not examined in this study also could be relevant factors in affecting nest temperatures, including phenology [78–80], substrate type [81,82], substrate moisture [42,58,83,84], slope of a nest site [25,85], nest depth [49,86] or proximity to other nests [87].

Regardless of the roles of other variables in nest-site choice in *C. picta*, the differences in nest canopy cover did not follow a strongly predictable latitudinal pattern, in contrast with findings from other studies of geographical variation in nest-site choice in reptiles (*Chelydra serpentina* in [53], *Physignathus lesueurii* in [54]). These differences between our study and previous work could be attributed to methodological or species differences. For example, species potentially have different constraints on embryonic development and therefore pressures on nesting behaviours (e.g. [78]). Importantly, the aforementioned studies that examined geographical variation in nest-site choice used species with a very different pattern of TSD to that of *C. picta*, whereby females are produced at warm and cool incubation temperatures and males are produced at intermediate incubation temperatures. We nonetheless uncovered a conceivable latitudinal pattern of divergence between mean nest and representative site CTEs in *C. picta*, consistent with the idea that females in more northern locations select nest sites to minimize embryonic exposure to cooler temperatures.

Our results suggest that females generally identify nesting sites with less canopy cover, but the mechanistic basis for this behaviour remains unclear. Because these turtles initiate nesting diurnally, the mechanism has been hypothesized to be a visual cue whereby females ‘assess’ canopy cover [52], instead of immediate thermal [88] or olfactory [87] cues, which are more transient. Indeed, *C. picta* demonstrably learn to navigate changing ultraviolet landscapes [89]. Physiological underpinnings notwithstanding, these turtles exhibit repeatable nesting behaviour, with certain females consistently choosing specific canopy cover for their nests year-to-year [90]. This consistency renders nest-site choice a relatively reliable target for natural selection and, hence, for adaptive responses. Even so, the field heritability of nest-site choice is relatively low and context dependent [91] and is unexplored at five of our six locations, so swift microevolution is relatively unlikely. Regardless, if cues for identifying suitable nest sites become unstable predictors or mismatched to the environment in the context of climate change, the potential for adverse fitness consequences is considerable.

Nest-site choice is a critical life-history trait with the potential to affect maternal fitness and survival, embryo phenotypes and survival, and juvenile performance across oviparous taxa [11,16,92]. Thus, the ultimate advantages for nest-site choice vary among taxa (and even among populations) and are not necessarily mutually exclusive. As we show, choice of nest sites in wild *C. picta* minimally involves buffering embryos from experiencing current temperature-induced selection, along with maternal manipulation of offspring sex ratios [28] and other offspring traits [18,29], but probably not nest depth [86] or maternal predation risk [93]. Nest site choice might serve as an important mechanism by which some organisms could at least partially mitigate the effects of rapidly changing environmental conditions (reviewed in [7]). However, in organisms like *C. picta*, longer lifespans constrain microevolution [67], and both reduced microhabitat availability in certain locations [51] and intrinsic morphology (i.e. hind limb length; [86]) limit potential responses. Hence, shifts in nesting behaviour for turtles might be a weak mechanism to respond to rapid environmental changes (e.g. [94]).

Understanding how geographically widespread species have adjusted to contemporary environmental conditions can better enable researchers to predict how such species with environmentally sensitive traits might accommodate rapidly changing environments. In the face of global climate change [95] and other rapid and massive anthropogenic alterations of local environments (e.g. [96]), it is critical to understand if the current mechanism(s) of nest-site choice will be viable for organisms to employ to keep pace with these ongoing environmental shifts. As females currently choose nest sites that are relatively warm and sun exposed, a behavioural shift towards nesting in shaded areas might counter or minimize the effects of climate change. However, the repeatable nesting behaviour [90] and long generation times of *C. picta* might reduce the capacity of this behaviour to change rapidly enough to match the current pace of climate change. We found females are not nesting randomly and that maternal behaviour is homogenizing nest environments across the range to a specific thermal environment (approx. 2°C when examining CTE). This homogenization of thermal developmental environment may lead to the patterns observed in common-garden studies, in which geography and local thermal environment do not explain patterns of phenotypic variation [44,47]. Future research should examine the impacts of nest-site choice on different components of offspring fitness (e.g. [17,29]), as well as focus on examining the potential of maternal behaviours to buffer selection on embryo traits in ways that slow the pace of adaptation (*sensu* [4,33]). Such information will enable researchers to better predict [97] the short- and long-term consequences of nest-site choice in temporally and spatially changing environments. Addressing these issues in a variety of taxa is urgent given the widespread alterations in biotic (e.g. introduced species) and abiotic (e.g. flooding frequency) environments owing primarily to human activity.

## Data Availability

Data is available from the Dryad Digital Repository: https://doi.org/10.5061/dryad.rn8pk0p7p [98]. The data are provided in the electronic supplementary material [99].
